# Supplementary material for: Active site specificity profiling datasets of matrix metalloproteinases (MMPs) 1, 2, 3, 7, 8, 9, 12, 13 and 14
Source: Data Brief. 2016 Feb 22;7:299–310. doi: 10.1016/j.dib.2016.02.036 (PMC4777984; doi:10.1016/j.dib.2016.02.036)
Supplement: Supplementary file 10 — Supplementary material [file mmc10.zip › WebPICS_hMMP7_G_1%/subsite_coop.html]

 

PICS results


- # About
- # Manual
- # Analysis
- # Results

- Redraw
- Seqlogo
- Dependency
- Coop

**Potential subsite cooperativity analysis for hMMP7\_G\_1%**   
Minimum difference for subsite dependency was set to +- 10 percentage-points  
Subsite dependency was checked for all positional occurences above 1 x natural abundance  
**Subsite dependency check is restricted to P3 - P3'**

|  |  |  |  |
| --- | --- | --- | --- |
| Fixed residue | Affected residue(s) | Change (percentage-points) | Vice-Versa change |
| P3\_A | P2prime\_L | 12.7 | 20.4 |
| P3\_A | P2prime\_V | -11.0 | -15.3 |
| P3\_I | P2\_N | 17.0 | 15.7 |
| P3\_I | P3prime\_A | 41.1 | 17.6 |
| P3\_P | P1\_G | 11.4 | 15.2 |
| P3\_P | P1prime\_I | 23.6 | 11.4 |
| P3\_P | P2\_K | 10.2 | 11.6 |
| P3\_P | P3prime\_V | 10.8 | 13.3 |
| P3\_V | P1\_K | 10.4 | 14.7 |
| P3\_V | P1\_Q | 11.6 | 18.6 |
| P3\_V | P2prime\_T | 11.0 | 16.5 |
| P2\_A | P3prime\_K | 16.5 | 14.5 |
| P2\_C | P3prime\_C | 23.2 | 30.8 |
| P2\_H | P1\_C | 17.5 | 21.9 |
| P2\_K | P2prime\_I | 11.6 | 10.2 |
| P2\_K | P2prime\_R | 12.2 | 11.4 |
| P2\_K | P3prime\_S | 14.7 | 18.7 |
| P2\_K | P3prime\_V | 13.4 | 14.5 |
| P2\_L | P1\_P | 21.7 | 39.0 |
| P2\_L | P1\_R | 11.2 | 11.2 |
| P2\_M | P1\_H | 16.9 | 16.9 |
| P2\_M | P1prime\_M | 35.1 | 21.9 |
| P2\_N | P1\_G | 23.4 | 25.3 |
| P2\_N | P2prime\_R | 13.9 | 12.0 |
| P2\_N | P2prime\_T | 13.3 | 10.8 |
| P2\_Q | P1\_S | 27.8 | 21.9 |
| P2\_Q | P2prime\_L | 24.1 | 13.3 |
| P2\_Q | P3prime\_A | 28.3 | 11.2 |
| P2\_S | P1\_Q | 21.6 | 18.7 |
| P2\_S | P2prime\_Q | 16.4 | 19.3 |
| P1\_A | P2prime\_T | 12.4 | 14.0 |
| P1\_C | P2prime\_L | 62.7 | 12.5 |
| P1\_G | P2prime\_R | 15.8 | 12.6 |
| P1\_G | P2prime\_T | 15.2 | 11.4 |
| P1\_G | P3prime\_S | 18.3 | 19.9 |
| P1\_H | P1prime\_M | 35.1 | 21.9 |
| P1\_K | P1prime\_I | 26.9 | 13.8 |
| P1\_K | P2prime\_R | 14.3 | 16.3 |
| P1\_K | P3prime\_Q | 10.9 | 16.9 |
| P1\_N | P2prime\_I | 15.2 | 11.4 |
| P1\_N | P3prime\_N | 11.8 | 17.6 |
| P1\_P | P2prime\_L | 27.7 | 13.9 |
| P1\_Q | P2prime\_H | 10.8 | 40.8 |
| P1\_Q | P2prime\_Q | 13.3 | 18.1 |
| P1\_R | P2prime\_I | 18.0 | 20.2 |
| P1\_R | P3prime\_G | 27.2 | 25.8 |
| P1prime\_M | P3prime\_Q | 18.3 | 13.3 |
| P2prime\_I | P3prime\_G | 19.5 | 16.5 |
| P2prime\_Q | P3prime\_V | 19.3 | 16.4 |
| P2prime\_R | P3prime\_Q | 13.3 | 18.1 |
| P2prime\_T | P3prime\_V | 10.8 | 13.3 |

  
**PICS analysis of protease: hMMP7\_G\_1%**  
163 cleavage sites analyzed  
PICS library made with (T)rypsin, (G)luC or (C)hymotrypsin:   
Cutoff for graphic display: 2 x natural abundance  

|  |  |
| --- | --- |
| Positional occurences  (table for total and relative (in %) values) | Occurences relative to natural abundance (table) |
|  |  |
